# In situ modelling of biofilm formation in a hydrothermal spring cave

**DOI:** 10.1038/s41598-020-78759-4

**Published:** 2020-12-10

**Authors:** Dóra Anda, Attila Szabó, Petra Kovács-Bodor, Judit Makk, Tamás Felföldi, Éva Ács, Judit Mádl-Szőnyi, Andrea K. Borsodi

**Affiliations:** 1 Department of Microbiology, ELTE Eötvös Loránd University, Pázmány P. sétány 1/C, 1117 Budapest, Hungary; 2 Danube Research Institute, Centre for Ecological Research, Karolina út 29, Budapest, 1113 Hungary; 3 Department of Geology, ELTE Eötvös Loránd University, Pázmány P. sétány 1/C, 1117 Budapest, Hungary; 4 Faculty of Water Sciences, National University of Public Service, Bajcsy-Zsilinszky utca, 12-14, 6500 Baja, Hungary

**Keywords:** Biofilms, Microbial communities, Microbial ecology, Ecological modelling

## Abstract

Attachment of microorganisms to natural or artificial surfaces and the development of biofilms are complex processes which can be influenced by several factors. Nevertheless, our knowledge on biofilm formation in karstic environment is quite incomplete. The present study aimed to examine biofilm development for a year under controlled conditions in quasi-stagnant water of a hydrothermal spring cave located in the Buda Thermal Karst System (Hungary). Using a model system, we investigated how the structure of the biofilm is formed from the water and also how the growth rate of biofilm development takes place in this environment. Besides scanning electron microscopy, next-generation DNA sequencing was used to reveal the characteristic taxa and major shifts in the composition of the bacterial communities. Dynamic temporal changes were observed in the structure of bacterial communities. Bacterial richness and diversity increased during the biofilm formation, and 9–12 weeks were needed for the maturation. Increasing EPS production was also observed from the 9–12 weeks. The biofilm was different from the water that filled the cave pool, in terms of the taxonomic composition and metabolic potential of microorganisms. In these karstic environments, the formation of mature biofilm appears to take place relatively quickly, in a few months.

## Introduction

Thermal karst environments provide diverse habitats for microorganisms having planktonic or biofilm forming lifestyles. Attachments of microorganisms to natural or artificial surfaces (e.g., rocks, glass slides) and the development of biofilms are rather complex processes which can be influenced by several factors^1^. Nevertheless, our understanding of these systems is incomplete to date, only a few studies have focused on the bacterial metagenome of cave speleothems^2–4^. Nitrogen-based primary production strategy was detected based on the metabolic profiling of the speleothems of the Weebubbie Cave (Australia) and Kartchner Caverns (Arizona, USA)^2,4^. However, metagenomic study of highly acidic (pH 0–1) snottites showed that *Acidithiobacillus* species, which made up more than 70% of the community, were involved in the primary production in the Frasassi cave (Italy). It has also been confirmed that these organisms use the Sox system for the oxidation of reduced sulfur compounds^4^.

In the last decade, several microbiological studies have been conducted in the Buda Thermal Karst System (BTKS) which is one of the few hydrogeological systems in the world where springs, caves and the effects of hydrothermal fluids on carbonate rocks can be recently examined. Studying the prokaryotes inhabiting the BTKS have been brought new insight into the microbially influenced hypogenic speleogenesis^5–8^. All these studies were based on samples from spring caves. The presence of complex meso- and thermophilic biofilm microbial communities based on the activity of a special and interconnected autotrophic sulfur and nitrogen prokaryotic metabolic network can be assumed in the studied systems^5–8^. However, the phases of biofilm formation, the potential influencing factors and the duration of biofilm maturation is not yet clarified.

Therefore, the aim of this study was to monitor the biofilm development in the quasi-stagnant thermal water of Rudas-Török (RT) spring cave (a part of the BTKS) using an in situ experimental model system. High-throughput next-generation DNA sequencing was used to reveal the characteristic taxa and major shifts in the composition of the bacterial communities, and electron microscopy was applied to observe the morphological changes in the structure of biofilms. As a result, the succession of biofilm formation was revealed under controlled conditions. Site-based long-term monitoring of the cave biofilms, the planktonic bacterial community and the physical and chemical parameters of the pool water provide new information on biofilm formation in thermal karst systems.

## Materials and methods

### Description of the sampling sites

**Figure 1:**
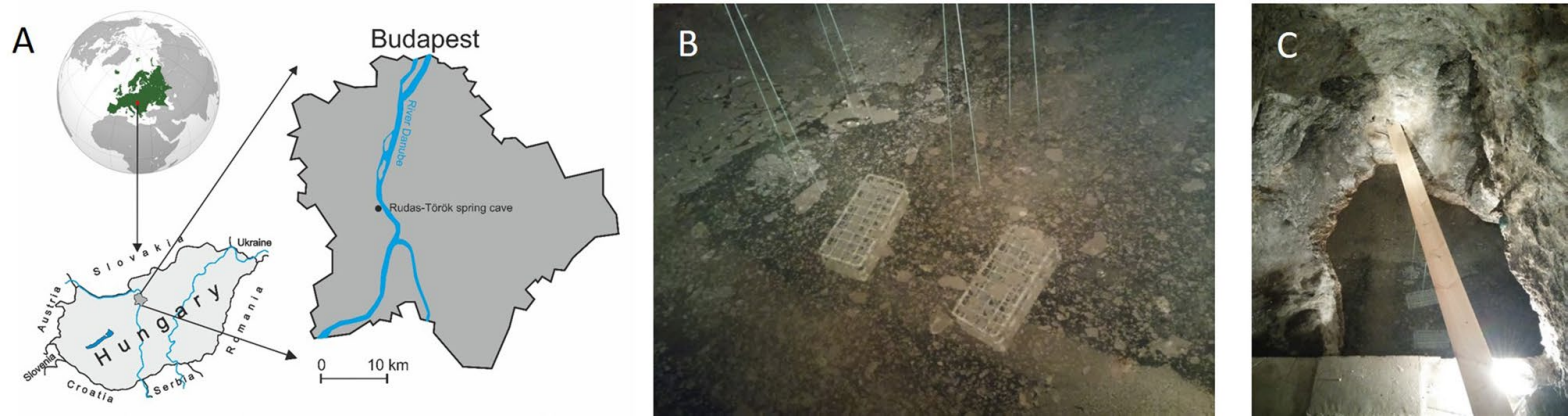
Geographic location of the sampling site (**A**). The arrangement of the in situ experimental model system in the RT spring cave (**B**,**C**).

The RT spring cave is located in the southern part of the BTKS close to the Danube (Fig. 1A) and offers an ideal opportunity to conduct an in situ model experiment. Here, the discharged thermal fluids mainly consist of upwelling thermal waters mainly meteoric origin karst water with less, additional saline basinal fluids^9^. The chemical parameters of the thermal spring are stable and influenced only by the water level of the Danube^10^. The water level in the spring cave is controlled also by an overflowing system and quasi-stagnant.

In order to explore the formation of microbial biofilm under natural conditions, an in situ model system was set up in the RT spring cave (Fig. 1B). The most appropriate place for our experiment was the cave pool (size: 3 m long × 2 m wide). It is an easy-to-access, undisturbed environment and closed from the public visitors. A wooden slat was placed over the approximately 0.5 m deep pool about 30 cm and surface disinfected polypropylene test tube racks containing sterile glass slides were fixed on it using fishing line (Fig. 1C). For the biofilm formation, glass slides were immersed into the water-filled cave pool 10 cm below the water surface. Glass slide samples were taken aseptically every three-weeks during the 30 weeks of sampling period, in three replicates. A pool water sample from the Rudas–Török spring cave (RTW) was also taken at the beginning of the experiment. The RTW sample was collected 10 cm below the water surface from the immediate vicinity of the glass slides without agitation the pool water before sampling. An additional biofilm sample was taken one year after the start of the experiment with three replicates The glass slides collected during the sampling were separately immersed into bottles containing sterile water and transported to the laboratory under refrigerated conditions. Samples were stored at 6–8 °C until being processed in the laboratory within 4 h.

### Determination of physical and chemical parameters of the water

During the in situ experiment, the pH, temperature, specific electrical conductivity and dissolved oxygen concentration of the pool water were measured using a WTW MultiLine P 8211 multi-parameter portable meter next to the test tube racks containing the glass slides. In addition, specific electrical conductivity, temperature and water level was measured continuously by a DATAQUA datalogger and was compared with the actual level of the Danube river. All other parameters were measured in the laboratory as previously described^6,7^.

### Scanning electron microscopy

To study the biofilms developed on the glass slides, approx. 1 × 1 cm^2^ quadrants were cut for scanning electron microscopy (SEM) with a glass cutter and placed in glutaraldehyde (5% in 0.1 M phosphate buffer) for 3–4 h at room temperature. All subsequent steps were the same as previously described in Anda et al.^7^. The samples were examined using a Zeiss EVO MA 10 scanning electron microscope at an accelerating voltage of 10 kV.

### Next-generation amplicon sequencing

Approximately 1000 ml of the water sample was filtered through a 0.22 μm pore diameter polycarbonate filter (Millipore). The biofilm samples were removed from the glass slides with sterile razor blades into sterile water and compacted by centrifugation (15,000×*g* for 2 min, centrifuge: Z33MK-2 (Hermle, Germany)). Total genomic DNA was extracted from the samples using Ultra Clean Soil DNA Isolation Kit (MO BIO Laboratories Inc., Carlsbad, CA, USA) according to the manufacturer’s instructions with the exception that cell disruption step was carried out by shaking at 30 Hz for 2 min in a Mixer Mill MM301 (Retsch, Haan, Germany).

The PCR amplification and sequencing of the 16S rRNA gene, the subsequent bioinformatic analysis and taxonomic assignments were performed as described in detail by Szabó et al.^11^, with the exception of taxonomic assignment which was made using the ARB-SILVA SSU 138 database as reference^12^. Analysis scripts are also available as Supplementary Material of the aforementioned paper. To obtain the same number of sequences per sample for beta diversity evaluations and to compare alpha diversity, subsampling sample reads to the smallest data set (n = 2954), calculation of richness (sobs—number of OTUs at a 97% similarity threshold, Chao1—Chao 1 index and ACE—Abundance-based Coverage Estimator) and diversity estimators (Simpson’s inverse and Shannon indices) using 1000 iterations were executed using the mothur software^13^.Non-metric multidimensional scaling (NMDS) ordination was carried out in R^14^ using the ‘vegan’ package^15^, SIMPER test was performed using the Past3 program^16^. Raw sequence data can be accessed under the NCBI BioProject ID PRJNA483930.

## Results

### Physical–chemical parameters of the pool water in the spring cave

During the one year monitoring period, the temperature of the water in the Rudas-Török spring cave ranged from 34.3 to 38.2 °C (Suppl. Fig. S1). Continuous decline in temperature was observed from late September 2015 to late January and an increasing trend was detected from the end of January until September 2016.

The specific electrical conductivity was 1715.1 ± 7.2 µS cm^−1^. The water level in the spring cave was an average 0.5 ± 0.01 m, while the water level of River Danube was 231.6 ± 1.08 m a.s.l. (Suppl. Fig. S1).

The accuracy for the water level is ± 0.1%, for the temperature is 0.1 °C and for the specific electrical conductivity is 1%.

The pH and dissolved oxygen content of the cave water is shown in Suppl. Fig. S2. The pH values were between 6.0 and 6.6 during the measurement period, while the dissolved oxygen content ranged between 0.2 and 1.2 mg L^−1^.

Relatively high sulfate (309 mg L^−1^) concentration was measured in the RT spring cave’s water taken at the beginning of the experiment. The total organic carbon content and the total nitrogen concentration of the thermal water was 6.2 mg L^−1^ and 0.9 mg L^−1^. Concentrations of NH_4_^+^-N, NO_3_^–^N, and NO_2_^–^N were 0.2 mg L^−1^, < 1 mg L^−1^, and 0.2 mg L^−1^, respectively. These results were also related to the values previously reported by Borsodi et al.^6^ and Enyedi et al.^17^.

### Scanning electron microscopic observations of biofilm structure

**Figure 2:**
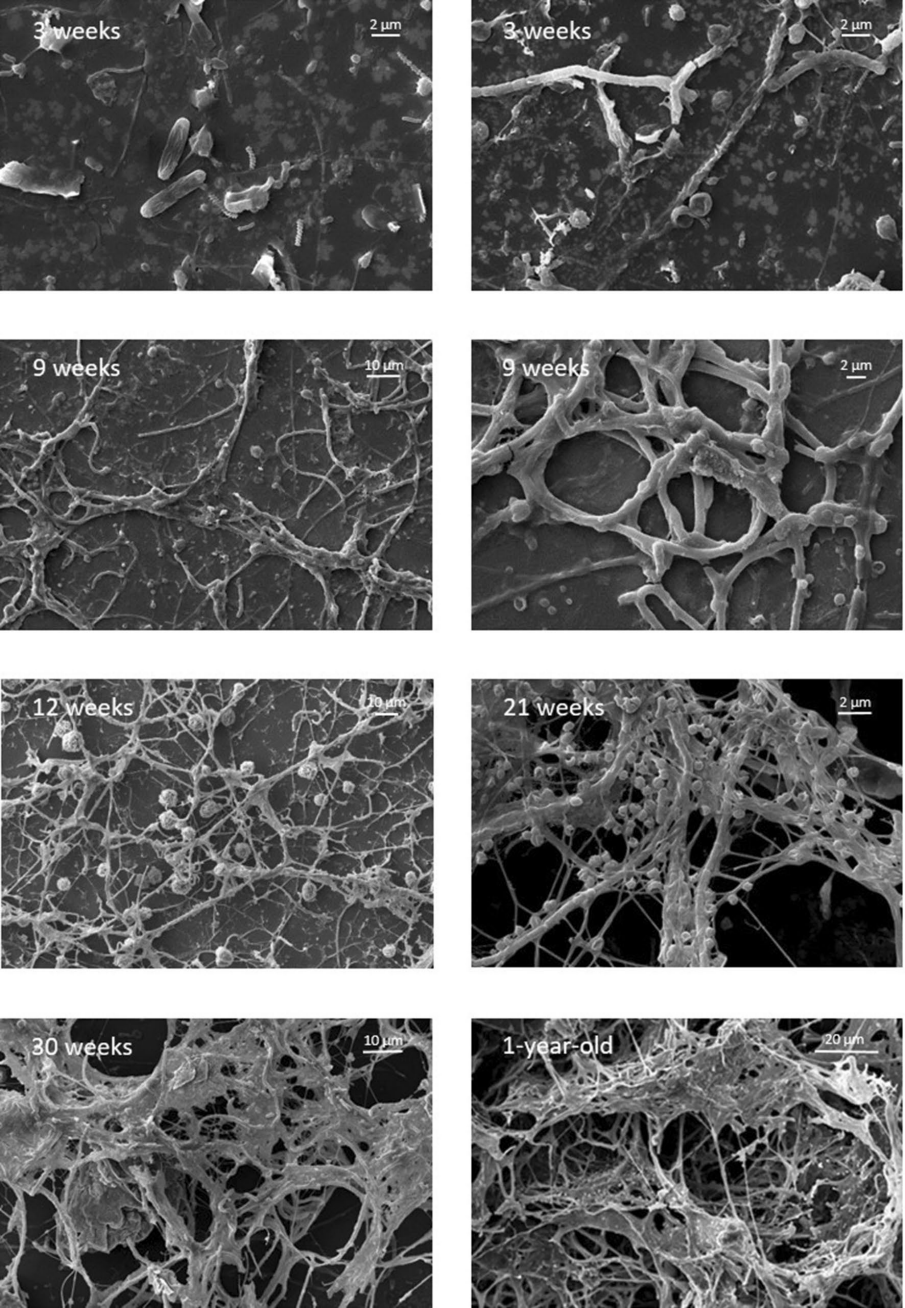
SEM micrographs of biofilm samples developing on the glass slides during a one year period (3 weeks (**A**,**B**), 9 weeks (**C**,**D**), 12 weeks (**E**), 21 weeks (**F**), 30 weeks (**G**), 1-year-old biofilm (**H**)) in the RT thermal spring. *Scale bar* (**A,B,D,F**) 2 µm, (**C,E,G**) 10 µm, (**H**) 20 µm.

Only few, filamentous bacteria with different lengths, cocci, rod- and spiral-shaped bacteria, typical of *Nitrospira* were observed on the high-resolution SEM images of the three-week biofilm sample (Fig. 2). An increasing number of cells with predominantly filamentous morphology were detected in the case of the six and nine-week biofilm samples (Fig. 2). In addition to filamentous bacteria of various thickness, cell aggregate-forming rods and cocci of different size embedded in (most probably) calcium carbonate minerals accumulating exopolysaccharides were also observed. These/such minerals were also well visible on the SEM image of twelve-week biofilm samples (Fig. 2). The cells also occurred in embedded mucous material. This mucus, which appeared on the six-week biofilm sample for the first time, was the extracellular polymer matrix (EPS) secreted by the biofilm forming bacteria. The multilayer network architecture of microbial biofilm formed mainly by long, filamentous bacteria can be seen on the twenty-one and twenty-four-week biofilm images (Fig. 2).

Detected cells exhibited predominantly filamentous morphology with different thickness and appearance and minerals (e.g., rhomboidal calcite) were embedded in large amounts of mucous material on the SEM images of twenty-seven, thirty-week and one-year biofilm samples (Fig. 2).

### Bacterial taxonomic composition during biofilm maturation

Pyrosequencing approach based on the variability of the V3-V4 region of the 16S rRNA gene was used to reveal the characteristic taxa and major shifts in the composition of the developing biofilm bacterial communities. A total of 88,000 high-quality bacterial 16S rRNA gene sequences were obtained from the 12 samples (Table 1).

**Table 1:** Bacterial species richness (ACE and Chao1) and diversity indices (inverse Simpsons and Shannon–Wiener) calculated from NGS data.

Sample ID	No. of sequences^a^	Sobs	Chao1	ACE	Shannon	Inverse Simpson
RTW	2954 (6536)	46 ± 2.5	59 ± 8.2	65 ± 10.7	0.4 ± 0.02	1.1 ± 0.01
3 weeks	2954 (2954)	143	162	173	3.5	17.1
6 weeks	2954 (9010)	176 ± 5.3	240 ± 21.8	266 ± 37.9	3.3 ± 0.03	10.5 ± 0.3
9 weeks	2954 (5986)	198 ± 4.9	260 ± 20.7	273 ± 27.9	3.8 ± 0.02	17.4 ± 0.5
12 weeks	2954 (7291)	172 ± 5.6	243 ± 24.4	265 ± 39.9	3.6 ± 0.02	17.4 ± 0.4
15 weeks	2954 (13,152)	190 ± 6.2	269 ± 24.9	287 ± 40.2	3.6 ± 0.03	14.1 ± 0.5
18 weeks	2954 (8225)	193 ± 5.7	273 ± 24.9	320 ± 49.7	3.7 ± 0.02	17.8 ± 0.5
21 weeks	2954 (8555)	199 ± 6.3	288 ± 28.4	360 ± 52.5	3.7 ± 0.03	14.6 ± 0.5
24 weeks	2954 (5102)	204 ± 4.6	263 ± 18.4	302 ± 37.8	3.8 ± 0.02	18.3 ± 0.5
27 weeks	2954 (4800)	202 ± 4.5	253 ± 15.7	266 ± 14.6	3.8 ± 0.02	18.9 ± 0.4
30 weeks	2954 (8352)	216 ± 6.5	311 ± 28.3	351 ± 51.2	4.0 ± 0.02	25.6 ± 0.7
1 year	2954 (3431)	187 ± 2.2	213 ± 6.9	222 ± 6.1	3.9 ± 0.01	23.7 ± 0.3

^a^Numbers in parentheses stand for the total number of sequences obtained with NGS; for calculating richness estimators and diversity indices, read numbers were subsampled to the read number of the sample having the lowest sequence count.

The values of the species estimation indices such as the number of OTUs, Chao1 and ACE (frequency-based coverage estimation index) were the lowest in the water sample, and in the third-, sixth- and twelfth-week biofilm samples (Table 1). The highest number of OTUs was observed in the 30^th^ week biofilm sample. Both Shannon and inverse Simpson diversity indices were the lowest in the water sample, while their values showed more or less increasing tendency during the maturation of the biofilm. The three-week biofilm was made by 143 OTUs, the 30-week biofilm by 214, and the one-year biofilm by 187 OTUs. In contrast, 47 OTUs were found in the water sample. Only 8 OTUs were shared between the 3 and 30 week, one-year-old biofilm and thermal water samples, and an additional 20 OTUs were shared among the biofilm samples (Suppl Fig. S3). Many OTUs were specific to only one sample, only to the developing or only to the mature biofilm.

**Figure 3:**
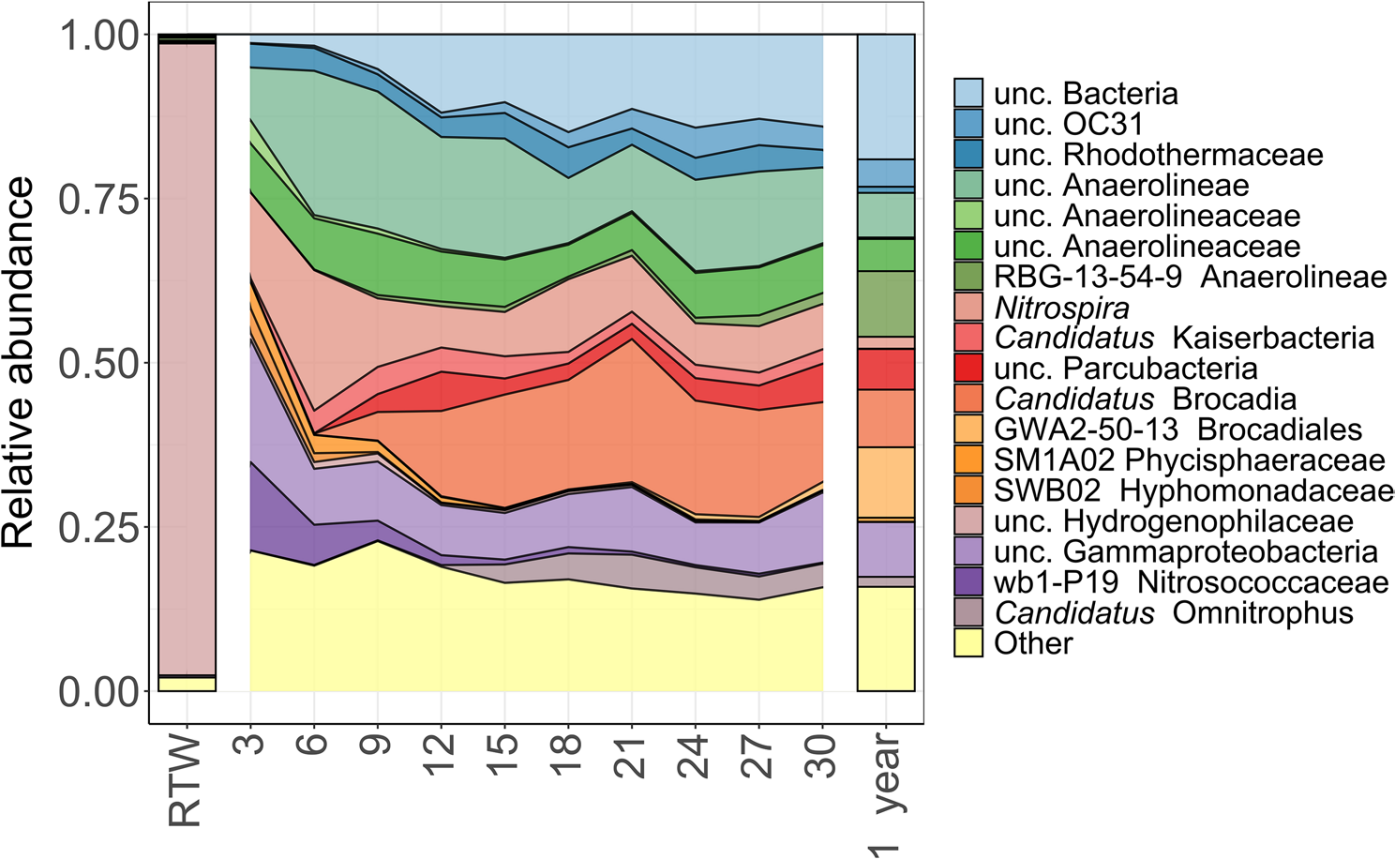
Percentile distribution of amplicon sequences on genus level revealed from the RTW sample and changes in the bacterial composition of biofilm communities in the one year period based on the 16S rRNA gene amplicon sequence data. Genera having < 2% relative abundance are combined in the “Other” category.

The reads were classified into altogether 40 different bacterial phyla in the water and biofilm samples. The taxonomic diversity at phylum level reached the maximum in the third week of the biofilm formation. Members of the phyla Chloroflexi, Patescibacteria, Planctomycetota, Proteobacteria and Nitrospirota, Bacteroidota and an unclassified group of Bacteria proved to be most abundant in the biofilm samples, but their relative abundance changed at different rates during the one year period. Some genus level bacterial taxa (e.g., unclassified Anaerolineaceae) were abundant throughout the whole studied period, while the abundance of others (e.g., unclassified Parcubacteria, *Candidatus* Brocadia, unclassified Bacteria) fluctuated (Fig. 3).

The phylum Chloroflexi dominated the biofilms during the whole studied period with an average of 23.9% relative abundance. The relative abundance of an unclassified Anaerolineaceae (Chloroflexi) increased from 8.1 to 21.9%. *Nitrospira* (Nitrospirota) proved to be the most abundant genus (21.4%) in the sixth week, while its relative abundance was only 1.8% in the one-year biofilm sample. During the studied period, the abundance of the phylum Patescibacteria showed a slight increase, although the highest relative abundance of the phylum (14.3%) was detected in the twelfth week. The relative abundance of this phylum Planctomycetota showed a significant increase over one year, while the members of the phylum were almost completely absent for the first six weeks. *Candidatus* Brocadia (Planctomycetota) was the most dominant taxon (21.7%) at the genus level in the twenty-first week. Several phylotypes of the phylum Proteobacteria were found in the biofilm samples, however, the relative abundance has significantly changed at the class level in the examined period. The most noticeable change was detected in the case of the Gammaproteobacteria. The representatives of the class were dominant in the initial period and their proportion decreased significantly during the year. In the biofilm samples, their relative abundance of reads representing unclassified Bacteria ranged between 1.3% and 19%, and their proportion increased in parallel with the maturation of the biofilm.

The water sample of the spring cave was dominated by an unclassified Hydrogenophilaceae (96.1% relative abundance). OTUs assigned to Hydrogenophilaceae were also detected in some of the biofilm samples but only with low relative abundance (0.1–1.2%). It was even more interesting that only one OTU (OTU3—unclassifiedHydrogenophilaceae) was present almost exclusively in the water sample. This OTU showed the highest sequence similarity with bacterial species characterized by sulfur oxidation metabolism (93.4% sequence similarity with *Sulfuritortus calidifontis*). The partial 16S rRNA gene sequence was compared with EzBioCloud database entries^18^.

**Figure 4:**
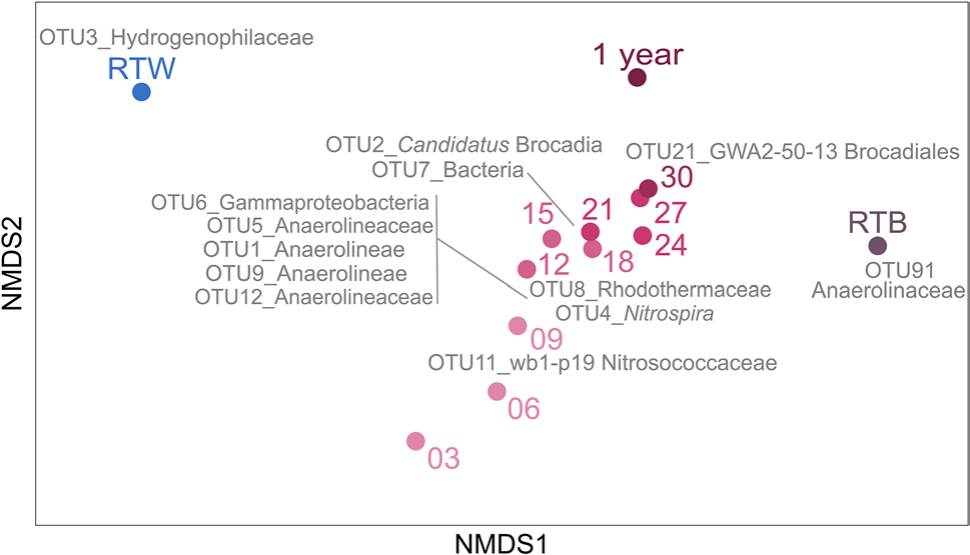
NMDS ordination based on Bray–Curtis distance of the bacterial OTUs from biofilm developed for years on the rock (RTB), glass slides (3–30 week and 1 year) and water samples (RTW) of the RT spring (stress: 0.03). Based on SIMPER analysis, OTUs responsible for 60% dissimilarity among samples are shown in gray. Figure is based on the combination the results of this study and Enyedi et al. (2019).

OTU91 (unclassified Anaerolineaceae) contributed the most to the sample type separation of the RTB sample (biofilm developed for years on the rock of the RT spring cave) based on SIMPER analysis, while the most abundant unclassified Hydrogenophilaceae (OTU3) in the pool water (Fig. 4). In the examined one-year period, the most dynamic changes were observed at OTU level. Certain OTUs were present only at the beginning of the studied period (OTU62—*Candidatus* Kaiserbacteria) or were only dominant in the first weeks, (OTU11—wb1-P19 (Nitrosococcaceae), OTU18—unclassified Gammaproteobacteria), other OTUs appeared only later in the biofilm (e.g. OTU2—Candidatus Brocadia, OTU7—unclassified Bacteria, OTU10—unclassified Bacteria).

## Discussion

Microorganisms inhabiting biofilms form structurally and functionally well-organized communities where the interactions among the participants can be extremely complex^1^. The in situ model systems arranged in controlled, natural and quasi-stagnant physical–chemical conditions are important to understand the changes that take place in the development of biofilms. Therefore, the present study performed in the RT spring cave highlighted some interesting phenomena on biofilm formation in thermal karst systems.

In the initial stage of biofilm formation, primary colonizing microorganisms generally bind through specific and non-specific interactions to a conditioning film adhering to solid surfaces, consisting of various organic materials^19^. It was also clearly visible on the SEM images (Fig. 2) made at the beginning of the model experiment. In the next stage of biofilm formation, microbial cells embed into an EPS. The increasing EPS production was observed from the 9–12 weeks during the experiment of biofilm development in the RT spring cave. The EPS consists of polysaccharides, glycoproteins, exoenzymes and nucleic acids that attach directly to the surface, also known as a substrate^20,21^.

The microscopically observed biofilm maturation correlated well with species richness estimators and diversity indices. The number of OTUs greatly increased during the first nine weeks when the morphological diversity of the biofilm also became more and more complex. Most of the dynamic changes observed at the OTU level happened until the twelfth week, so we could state that nine to twelve weeks were needed for the maturation of the biofilm. The greatest difference between the species richness estimators was among the water and biofilm samples.

In an earlier study, a natural biofilm sample (RTB) developed for years on the rock of the RT spring cave^17^ was analyzed. The taxonomic composition of bacteria inhabiting the natural and experimental biofilms was similar. The most abundant community members of the RTB as well as the 3–30 weeks and one-year in situ model biofilm samples were also the same, showing no difference or selection between the glass slide and the carbonate rock as a surface substrate. Based on our previous results^17^, unforeseen taxonomic bacterial diversity was obtained from these highly radioactive environments (600 ± 21 Bq/L radon concentration in the case of Rudas-Török spring cave) based on next-generation sequencing data, containing mainly unclassified bacteria affiliated with low level similarity to cultured bacterial taxa. The surprisingly high diversity suggests that the microorganisms living here are well adapted to this extreme environment.

As regards the taxonomic diversity, Proteobacteria was detected with relative high abundance both in the water and biofilm samples (Fig. 3). It could be presumed based on the previous studies, because members of this phylum were frequently detected as constituents of bacterial communities in different cave samples^6,22–26^. The water sample of the RT spring cave was dominated almost exclusively by an unclassified Hydrogenophilaceae (OTU3) that showed the highest sequence similarity with chemo-lithotrophic sulfur-oxidizing bacteria (Fig. 3). OTU3 (with its 96.1% relative abundance) could be considered as the potential ‘core OTU' of the thermal water. To our knowledge, this is the first report about such extraordinary high relative abundance of an OTU in the thermal waters of karst caves. Deja-Sikora et al.^27^ reported a similar phenomenon, the dominance of one unclassified Betaproteobacteria OTU affiliated with *Comamonadaceae* (abundance ranging from 1.7 to 57.8%) in sulfide-rich waters of the Carpathian Foredeep. The authors assumed that members of *Comamonadaceae* were likely to represent archetypal microbial species in those waters. The dominant read of OTU3 originated from the discharging deep thermal waters, however, was almost completely absent from the biofilm samples of the spring cave. This finding was surprising because the role of bacteria arriving with the discharging water sample was hypothesized in the formation of the biofilm. Among Proteobacteria, an unclassified Gammaproteobacteria was the most abundant in the biofilm samples (Fig. 3), nevertheless, no more information is known about these unclassified phylotypes.

The phylum Chloroflexi was dominant in the biofilms throughout the studied period with the members of unclassified Anaerolineaceae (Fig. 3)*.* These Gram-negative, filamentous, thermophilic and strictly anaerobic, chemo-organotrophic organisms^28^ can serve as the basis of the biofilm formation in the RT spring cave. The non-cultivated members of the class Dehalococcoidi have fermentative metabolism and can use *N*-acetylglucosamine under anoxic conditions (using nitrate as electron acceptor). N-acetylglucosamine, which forms the backbone of the murein of most bacterial cell walls, is released continuously when cells are destroyed^29^. Presumably, the representatives of these filamentous Chloroflexi can be the first adherent organisms according to the SEM images (Fig. 2), and the low oxygen level in the spring cave may have favored their reproduction. The OTUs assigned to the phylum Chloroflexi were also frequent not only in the biofilm formed on the glass slides but in the biofilm developed on the rock surface of the RT spring cave as well^17^.

Representatives of the genus *Nitrospira* (Nitrospirota) were also present throughout the experiment. Their highest proportion were observed in the sixth week of the biofilm formation (Fig. 3). The characteristic cell shape typical for the genus *Nitrospira* has been observed in the three-week biofilm sample on the SEM images, as well (Fig. 2). Nitrogen is frequently a limited nutrient source in caves; therefore, the importance of the nitrogen cycle has been emphasized in other studies^25,30^. Chemolithotrophic autotrophic prokaryotes, including nitrifiers, play a key role in the primary production of cave environments^31^. The presence of ammonia-oxidizing *Nitrosospira* and nitrite-oxidizing *Nitrospira* and *Nitrobacter* were revealed previously from the deposits of the cave wall of the western Loess Plateau of China^32^ and the presence of these organisms were detected in the caves and spring caves of the BTKS^5–8^, as well. Through their activity, nitrite-oxidizing aerobic chemolithotrophic bacteria may contribute to the low nitrite concentration values, which were also measured in the cave waters of the BTKS.

A possible reason for the low ammonia content in the BTKS is the oxidation of ammonia, in which members of both the Archaea and Bacteria may be involved. The ammonia-oxidizing archaea (AOA) organisms belonging to the phylum Thaumarchaeota appeared in high proportion in the archaeal clone libraries created from biofilms originated from the caves and spring caves of the BTKS^6,7^. For the members of the Archaea, an increasing temporal trend was observed in the biofilms from the fifteenth weeks, although the primer-pair which was used for amplification is rather Bacteria-specific^33^. The diversity and importance of Archaea in karst cave environments, in contrast to the Bacteria, is largely unexplored^2,3,34^. In the study of the speleothems of the Weebubbie Cave (Nullarbor karst, Australia) and Kartchner Caverns (Arizona, USA), the authors also demonstrated the importance of members of Archaea, especially the ammonia-oxidizing Thaumarchaeota^2,3,34^. Our findings may confirm the hypothesis that AOA organisms could have an important role in the nitrification process in the RT spring cave as well.

The members of the phylum Planctomycetota (*Candidatus* Brocadia) proved to be dominant in the biofilm samples (Fig. 3). Representatives of the ‘*Candidatus* Brocadia’ may participate also in the local nitrogen cycle by the anaerobic oxidation of ammonia (anammox) combined with nitrite reduction that results in the formation of elemental nitrogen^35^. The anaerobic ammonia-oxidizing bacteria grow very slowly, the fastest growing species also have a 10-day generation time^36^, which may be associated with the fact that the relative abundance of the phylum showed a significant increase only from the sixth week of biofilm formation.

Representatives of the phylum Patescibacteria (unclassified Parcubacteria) were found in high proportions in the biofilm samples (Fig. 3). These organisms were mostly observed in anoxic environments^37^, their presence can be associated with the low dissolved oxygen values in the RT spring cave. The members of the Parcubacteria have small genome size (< 1.1 Mbp) and based on this, it can be assumed that they form symbiotic relationships in the biofilm^38^.

Unclassified bacterial reads were also occurred in a high proportion in the biofilm samples and their relative abundance showed a significant increase during a year. In a metagenomics study of carbonate caves in the Kartchner Caverns, pyrosequencing resulted nearly 400,000 partial 16S rRNA sequence data in the case of the 10 examined samples. Unfortunately, most of the taxa obtained from the cave could not be identified, whereas the vast majority of prokaryotic taxa in the absence of a cultured representative is still unknown to science^2^.

In conclusion, the in situ experiment performed in this study allowed us to examine the development of biofilm over a year under controlled but natural and near constant physical–chemical conditions. From the adhesion of the first microbial cells, biofilm differed fundamentally from the pool water of the spring cave. At least nine weeks were needed for the development of a mature biofilm regarding the morphological complexity and taxonomic diversity. The prokaryotes involved in the aerobic and anaerobic nitrification processes were characteristic in the biofilm samples, in addition to the anaerobic fermentative and filamentous Chloroflexi, in contrast to the water sample where the dominance of an uncultivated member of the family Hydrogenophilaceae was observed.

## Supplementary Information

Supplementary Information.

## Data Availability

Raw sequence data were submitted to NCBI under BioProject ID PRJNA483930.
