# Supplementary material for: In situ modelling of biofilm formation in a hydrothermal spring cave
Source: Sci Rep. 2020 Dec 10;10:21733. doi: 10.1038/s41598-020-78759-4 (PMC7729855; doi:10.1038/s41598-020-78759-4)
Supplement: Supplementary file 1 — Supplementary Information. [file 41598_2020_78759_MOESM1_ESM.pdf]

# In situ modelling of biofilm formation in a hydrothermal spring cave

Dóra Anda[1], Attila Szabó[1], Petra Kovács-Bodor[3], Judit Makk[1], Tamás Felföldi[1], Éva Ács[2,4], Judit Mádl-Szőnyi[3], Andrea K. Borsodi[1,2]

[1]Department of Microbiology, ELTE Eötvös Loránd University, Pázmány P. sétány 1/C, H-1117 Budapest, Hungary

[2]Danube Research Institute, Centre for Ecological Research, Karolina út 29, Budapest H-1113, Hungary

[3]Department of Geology, ELTE Eötvös Loránd University, Pázmány P. sétány 1/C, H-1117 Budapest, Hungary

[4]National University of Public Service, Faculty of Water Sciences, Bajcsy-Zsilinszky utca, 12-14, H-6500 Baja, Hungary

## Supplementary Figures

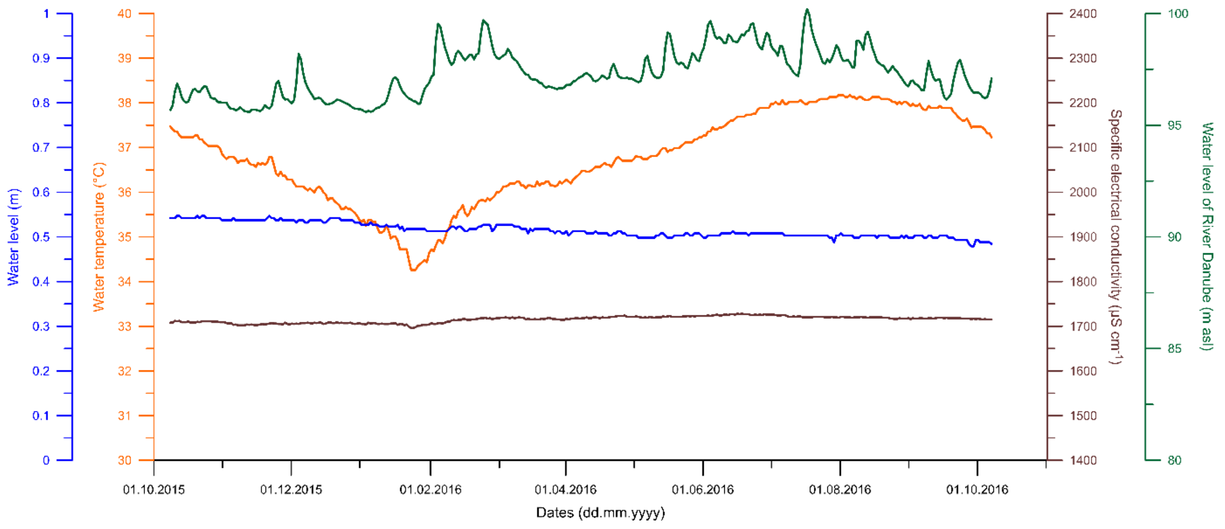


**Suppl Figure 1.** Changes in specific electrical conductivity, temperature and water level of RT spring cave compared with the actual level of the Danube river

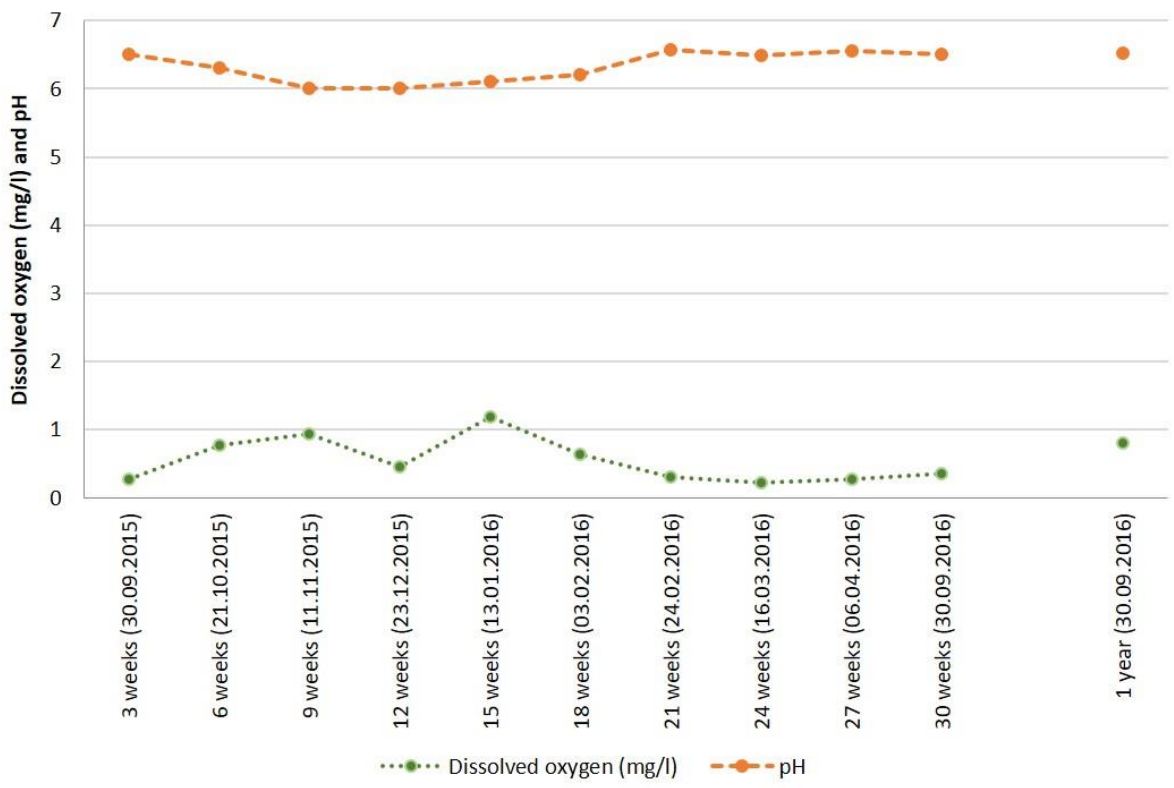


**Suppl Figure 2.** Changes in pH and dissolved oxygen content of RT spring cave water during the *in situ* experiment

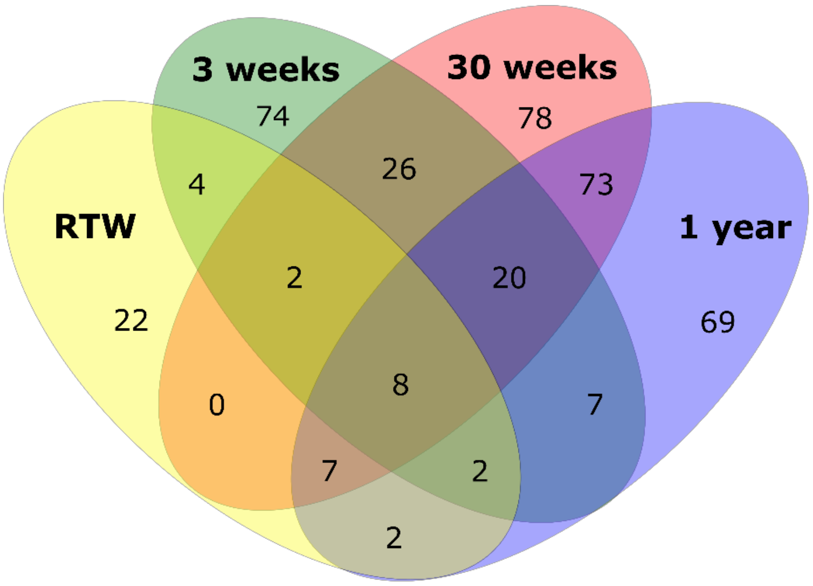


**Suppl Figure 3.** Venn diagram showing the unique and shared OTUs in 3 weeks, 30 weeks, one-year-old biofilm and thermal water samples
